# A Unified Approach to Nonlinear Transformation Materials

**DOI:** 10.1038/s41598-018-22215-x

**Published:** 2018-03-13

**Authors:** Sophia R. Sklan, Baowen Li

**Affiliations:** Department of Mechanical Engineering, University of Colorado Boulder, Colorado, 80309 USA

## Abstract

The advances in geometric approaches to optical devices due to transformation optics has led to the development of cloaks, concentrators, and other devices. It has also been shown that transformation optics can be used to gravitational fields from general relativity. However, the technique is currently constrained to linear devices, as a consistent approach to nonlinearity (including both the case of a nonlinear background medium and a nonlinear transformation) remains an open question. Here we show that nonlinearity can be incorporated into transformation optics in a consistent way. We use this to illustrate a number of novel effects, including cloaking an optical soliton, modeling nonlinear solutions to Einstein’s field equations, controlling transport in a Debye solid, and developing a set of constitutive to relations for relativistic cloaks in arbitrary nonlinear backgrounds.

## Introduction

**Figure 1:**
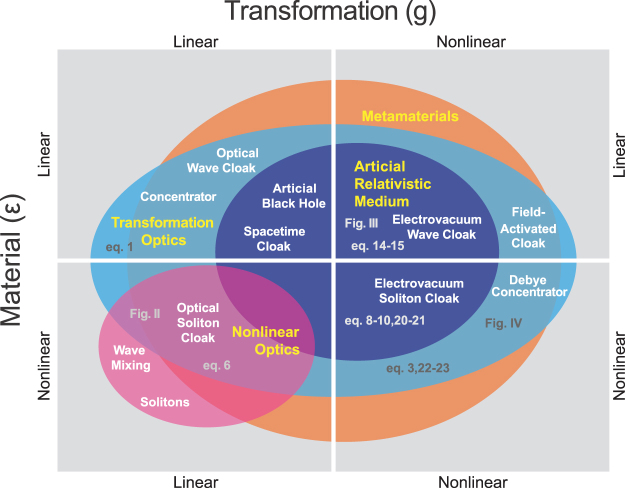
Representation of our transformation optics framework. Background material ($\epsilon$) and coordinate transform (*g*) can be linear or nonlinear with respect to applied fields, making four mutually exclusive cases. Within this parameter space, certain combinations satisfy transformation optics requirements (light blue ellipse). A subset of these also satisfy Einstein’s field equations (dark blue ellipse). When nonlinearity is included, effects from other fields, e.g. nonlinear optics (magenta circle) can become incorporated into transformation optics. Transformation optics is typically fabricated using metamaterials (orange circle), although other implementations are possible (if often trivial, e.g. lenses) and applications of metamaterials outside of transformation optics also exist. Examples of transformation optics devices from each quadrant are labelled, with the nonlinear examples being explored in the text (except the switchabe cloak, discussed in ref.^37^).

Transformation optics^1–9^, which uses geometric coordinate transformations derive the materials requirements of arbitrary devices, is a powerful technique. Essentially, for any geometry there corresponds a material with identical transport. With the correct geometry, it is possible to construct optical cloaks^10–12^ and concentrators^13^ as well as analogues of these devices for other waves^14–18^ and even for diffusion^19–26^. While many interpretations and formalisms of transformation optics exist, such as Jacobian transformations^4^, scattering matrices^27–31^, and conformal mappings^3^, one of the most theoretically powerful interpretations comes from the metric formalism^32^. All of these approaches agree that materials define an effective geometry, however the metric formalism is important since it allows us to further interpret the geometry. In particular, certain geometries correspond to solutions to Einstein’s field equations, which relate geometric curvature to gravitational forces. Materials that mimic these geometries, or artificial relativistic media, constitute a subset of transformation optics materials (dark blue circle, Fig. 1) that can effectively model relativistic effects^32^, such as black holes^33,34^ and gravitational lensing^35^ or create novel devices such as the space-time cloak (which hides events instead of objects)^36^.

One limitation of transformation optics, however, is the necessity of using linear materials (materials whose properties do not change with electric field, pressure, temperature, etc.). At present, the transformations that have been derived have exclusively been applied to linear media. That is, the focus has been upon media equivalent to an isotropic, homogeneous, linear background medium embedded in curvilinear coordinates. However, there is no necessity to maintain the constraint of linearity. In thermal transformations, researchers have already considered the case of temperature dependent transformations (which we shall generalize as “nonlinear transformations”), and shown how they are equivalent to a thermally nonlinear material embedded within a linear background^37,38^. However, considerations of background nonlinearity have thus far been absent. Moreover, nonlinear transformations lack the intuitive physical interpretation of linear transformation materials, where transport follows stationary geodesics. This intuition is useful when developing devices where geodesics bend and shift with the applied field.

In this paper, we shall present a unified theory of nonlinear transformation optics. We will consider both the case of a nonlinear background medium (bottom half of Fig. 1) and nonlinear transformations (right half of Fig. 1) in arbitrary combination. We shall begin by generalizing transformation optics theory to incorporate these nonlinearities, then consider examples illustrating this formalism from each of the new, nonlinear quadrants of Fig. 1. Examples will be selected for their practical significance, physical intuition, and clarity.

## Results

### Nonlinear Transformation Formalism

To begin, in linear transformation optics, the constitutive relation is^32^

1 $${\epsilon }^{ij}/{\epsilon }_{0}={\mu }^{ij}/{\mu }_{0}=\frac{\sqrt{-g}}{\sqrt{\gamma }}\frac{{g}^{ij}}{{g}^{00}}$$

where *g*^*ij*^ is the metric in transformed coordinates, *g* is the determinant of the metric, *g*^00^ is the time-like component of the metric (−1 for a static transform) and *γ* is the determinant of the untransformed metric (1 for Euclidean coordinates, *r*^2^ for cylindrical, etc.). That is,

2 $${g}^{i^{\prime} j^{\prime} }=\frac{\partial {x}^{i^{\prime} }}{\partial {x}^{i}}\frac{\partial {x}^{j^{\prime} }}{\partial {x}^{j}}{\gamma }^{ij}$$

where *γ*^*ij*^ is the metric of the reference frame (unprimed coordinates) and we have used Einstein summation notation for curvilinear coordinates (indices repeated as both subscript and superscript (covariant and contravariant) are summed, Latin indices are only over spatial dimensions, Greek indices are over space and time (0^*th*^) dimensions). Note that this formulation explicitly requires a stationary medium and observer. A fully covariant description is given in^39–42^ and is easily modified to incorporate the formalism developed here, but is less widely used than the stationary case.

#### Scalar field dependence

Since in most cases the variable of interest in a transformation materials problem is a scalar (temperature, pressure, electric potential) or can be approximated as such (electric field, fluid velocity, etc. for specific geometries), it is helpful to begin the consideration of field dependence by considering this scalar variable explicitly. By definition, this scalar variable is not changed by transformation media techniques except for the implicit change of spatial variable (e.g. *T*(*r*) → *T*(*r*′)). Moreover, scalars remain scalar even when raised to an arbitrary power, so rank of the tensors considered in equation 1 are not changed by the field dependence (even when they are Taylor expanded as a function of field strength). As the tensor rank governs the rules for the transformation, we conclude that equation 1 can easily be generalized to a nonlinear transformation of a nonlinear background by the relation

3 $${\epsilon }^{ij}(|\vec{E}|)/\epsilon (|\vec{E}|)={\mu }^{ij}(E)/\mu (E)=\frac{\sqrt{-g(E)}}{\sqrt{\gamma }}\frac{{g}^{ij}(E)}{{g}^{00}(E)}$$

where we have assumed that the nonlinearity is solely a function of implicitly time and coordinate-dependent electric field $|\vec{E}(\vec{r},t)|=E(\vec{r},t)=\sqrt{{E}^{i}(\vec{r},t){E}_{i}(\vec{r},t)}$ and position (henceforth an implicit depedence, with dependence upon other fields (e.g. magnetic field intensity, temperature, pressure, etc.) following trivially from this equation.

Note that the functional forms of $\epsilon (E)$ and *g*(*E*) are arbitrary. Assuming the coordinate transformation *x*^*i*^ → *x*^*i*′^ leaves Maxwell’s equations (or the corresponding equation of motion for other fields) unchanged, except for a change of variables (i.e.

4 $$ {\mathcal L} [E(x),{g}_{0},\epsilon (x,E(x)),x]= {\mathcal L} [E(x^{\prime} ),g(x^{\prime} ,E(x^{\prime} )),{\epsilon }_{0}(E(x^{\prime} )),x^{\prime} ]$$

for operator ${\mathcal L}$ that defines *E*), then the introduction of nonlinearity preserves transformation optics techniques, as the coordinates only enter the nonlinearity through the field. For example, this means that the Kerr nonlinearity (discussed in detail in the following section) should not be perfectly transformed according to the general rules for coordinate transformations. In particular, because that nonlinearity depends upon the electric field intensity $E(\vec{r},t)=\sqrt{{E}^{i}{E}_{i}}=\sqrt{{E}_{i}{g}^{ij}{E}_{j}}$ which is changes form under coordinate transformations, Maxwell’s equations are changed if the transformation is applied blindly^43,44^. If this effect is neglected, however, Maxwell’s equations actually obey the correspondence of equation 4 and $\vec{E}(\vec{x}) \rightarrow \vec{E}(\vec{x}^{\prime} )$, as desired.

#### Tensor field dependence

This scalar field nonlinearity is a useful form but is not the most general nonlinearity. In general, the field of interest will be an arbitrary rank tensor. However, if we assume that the functional dependence of *g*, $\epsilon$ are accurately represented by Taylor expansions in terms of field strength, then we need only consider the special case of a vector field depedence. Higher ranked tensors will result in terms which are effectively reducible to tensor products of vectors (e.g. ${\chi }_{ijkl}^{(T)}{T}_{kl}\leftrightarrow {\chi }_{ijkl}^{(E)}{E}_{k}{E}_{l}$). Because the rank of the tensor determines the properties under coordinate transformation, we thus see that the rules for the vector field dependent transformation cover the rules for any other tensor rank (assuming that the Taylor expansion holds). In particular, a material with anisotropic dependence upon the electric field (i.e. both strength and orientation) can be represented by the Taylor expansion

5 $$\epsilon (E)={\epsilon }_{ij}^{\mathrm{(0)}}+{\epsilon }_{0}{\chi }_{ijk}^{\mathrm{(2)}}{E}_{k}+{\epsilon }_{0}{\chi }_{ijkl}^{\mathrm{(3)}}{E}_{k}\,{E}_{l}\ldots $$

where ${\chi }_{ijk\ldots }^{\mathrm{(2}+)}$ is the nonlinear (electric) susceptibility (magnetic susceptibility can be defined similarly for *μ*(*H*)). Since each factor of *E*_*i*_ in the anisotropy corresponds to an additional scalar product,

6 $${\chi }_{ij{x}_{1}{x}_{2}\ldots {x}_{n}}^{(n)}=\prod _{a=1}^{n}\,{g}^{{x}_{a}{x}_{b}}{\chi }_{ij{x}_{1}{x}_{2}\ldots {x}_{b}\ldots {x}_{n}}$$

where indices *ij* are handled by the transformation of equation 3 which multiplies all factors of $\epsilon$. Note that this formalism can implicitly handle anisotropy in indices *ij*, as such linear anisotropy can be introduced as a transformation from an isotropic background, giving a composite transformation.

### Nonlinear Background – Linear Transform

In particular, if the nonlinearity takes the form of a Kerr nonlinearity

7 $${P}_{i}={\epsilon }_{ij}{E}_{j}-{\epsilon }_{(0)}{E}_{j}={\epsilon }_{(0)}({\chi }_{ij}^{(1)}{E}_{j}+{\chi }_{ijkl}^{(3)}{E}_{j}{E}_{k}{E}_{l})$$

(*P* is polarization and *χ* is susceptibility, which we assume to be isotropic), Maxwell’s equations remain unchanged under the cloaking transformation,

8 $$r^{\prime} =a+\frac{b-a}{b}r\mathrm{.}$$

**Figure 2:**
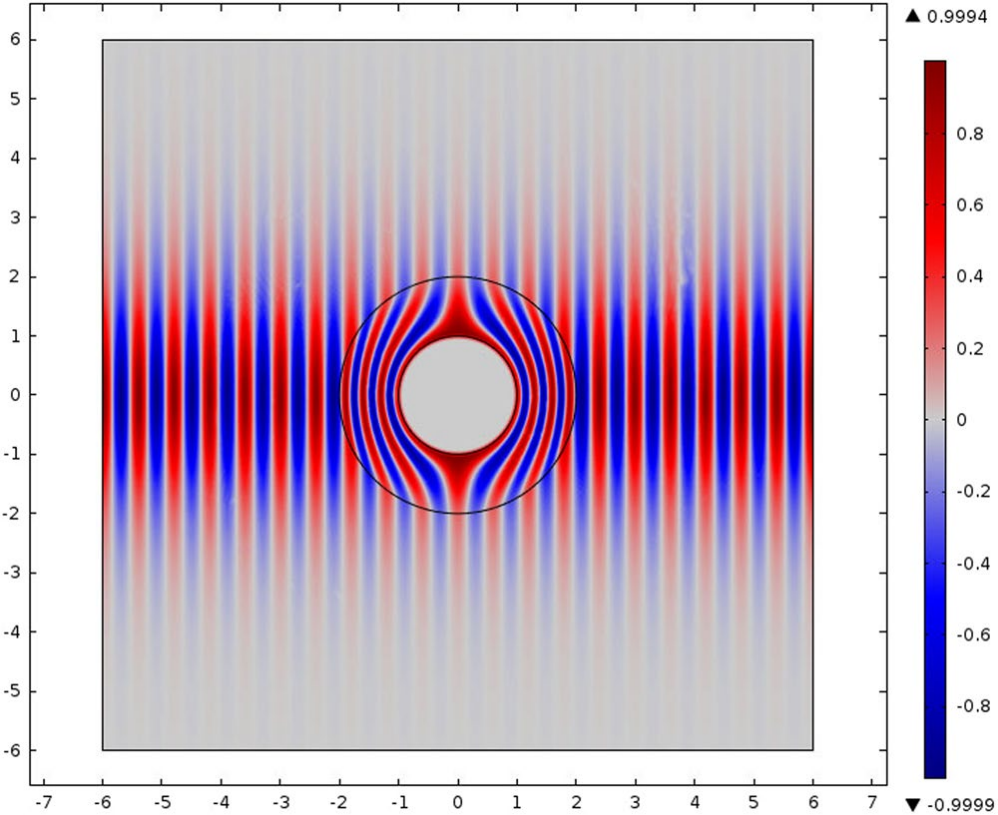
Cloak of a medium with Kerr nonlinearity. Note the variation in wave amplitude, corresponding to first spatial soliton mode.

Thus, if we can find a solution to Maxwell’s equations in Euclidean space with a Kerr nonlinearity, we can find a solution to Maxwell’s equations with a Kerr cloak permittivity (lower left in Fig. 1)

9 $${\epsilon }_{ij}={\epsilon }_{(0)}(1+{\chi }^{(1)}+{\chi }^{(3)}{E}^{2})[\begin{array}{cc}\frac{r-a}{r} & 0\\ 0 & \frac{r}{r-a}\end{array}]$$

by writing the Euclidean solution in primed (i.e. cloak) coordinates (note that we have taken advantage of the transformation *E*(*r*) = *E*(*r*′) and assumed that only one polarization of electric field is present^43^). (Including the full anisotropy of *χ*^(3)^, equation 9 is modified ${\chi }^{\mathrm{(3)}}|E{|}^{2}\to {\chi }_{ijkl}^{\mathrm{(3)}}{g}^{ka}{g}^{lb}{E}_{a}{E}_{b}$, more details are given in^43,44^) The Kerr nonlinearity is a special case of nonlinear optics with an exactly solvable system for special values of intensity *E*^2^ corresponding to optical soliton modes. For concreteness, we select the first spatial soliton^45^,

10 $$\vec{E}={A}_{0}\,{\rm sech} (y/{y}_{0}){e}^{i(\omega t-kz+\gamma z)}\hat{y},$$

where *A*_0_ is the soliton intensity, ${y}_{0}=|{A}_{0}|\sqrt{3{\chi }^{\mathrm{(3)}}}/2k$ is the pulse width, *k* is the wave-vector, *ω* frequency, $\gamma =3k{\chi }^{\mathrm{(3)}}|{A}_{0}{|}^{2}/4{n}_{0}^{2}$, and *n*_0_ is the linear index of refraction. Note that the polarization and direction of soliton propagation in this case are arbitrary, but because the equation 9 is isotropic with respect to these choices the system does not need to be modified for solitons of different polarization or propagation direction. (Higher orde solitons, however, will possess different combinations of frequencies and so will require some modification in practice for proper realization of the cloak.) The analytic solution is plotted in Fig. 2. Note that the cloaking is exact in the analytic case, despite the nonlinear background. While this solution is exact, it is limited to planar system (i.e. 1 + 1D) such as slab waveguides, since the Kerr spatial soliton is unstable in higher dimensions^46^. However, this approach is easily generalized to other systems with optical spatial solitons, including saturable Kerr nonlinearity, photoreactive materials, etc. Additionally, as discussed below, the spectral breadth of the soliton implies that the cloak cannot work perfectly for all the frequencies present (but can work perfectly for some and approximately for others, as the soliton spectrum is relatively narrow).

#### Applications

Moreover, while the ability to cloak a soliton is somewhat artificial, its success implies that an observer could not use a background Kerr nonlinearity to detect the presence of a nonlinear cloak, whereas a linear cloak would disperse the soliton and thereby render itself detectable. This could have applications in controlling waves in other nonlinear media with similar nonlinearities, such as protecting against rogue waves^47^ (although this is easier to accomplish in optics than surface waves, as the nonlinearity is more easily engineered there). In addition, nonlinearity is used to locate defects in acoustic/ultrasonic nondestructive testing^48–50^, including via acoustic solitons^51^, and to detect waves in sonar systems^52^. Applying this technique to an acoustic system, then, would prevent an observer from using the nonlinearity of a material to detect a cloak hidden within it. Since nonlinearities exist in most real materials (e.g. polycrystalline solids, porous media like clay or soil, and seawater and other complex fluids), the incorporation of nonlinearity to cloaking design is integral to rigorously prevent detection of buried objects or evade sonar. Similarly, a concentrator (equation 30) could be used to increase the density of a field in a region, thereby promoting soliton formation or promoting the interaction of multiple solitons.

#### Realization

Given that materials with a Kerr nonlinearity exist, it is likely that the simplest implementation of a soliton cloak is merely to incorporate such a material into existing metamaterial cloak designs. However, such cloak designs are based upon resonant effects (e.g. superluminal phase velocity at a single frequency) and therefore cannot be directly applied to multiple disparate frequencies. The lowest order spatial soliton, though, actually preserves the spectal properties of a plane wave fairly well. Compared to a plane wave in vacuum, there are only two modifications: the wave-vector along $\hat{z}$ is shifted from *k* to *k* − *γ* (i.e. still a single frequency) and additional components are introduced along *k*_*y*_ according to the Fourier transform of sech, which is sech(*πy*_0_*k*_*y*_/2). This is an approximately Gaussian spread centered at zero and with standard deviation 2/*πy*_0_. While this implies that the soliton only possesses frequencies in the neighborhood of the corresponding plane wave frequency, the Gaussian peak is an issue for perfect cloak operation. It is not, however, an impediment to imperfect cloaking. If, instead of mapping a finite region to a point, the scattering cross-section of a domain is reduced by a factor Δ*r*, then the resulting cloak is subject to the constraint

11 $$\frac{{\rm{\Delta }}\omega }{{\omega }_{0}}\le \frac{{\rm{\Delta }}r}{a}$$

where Δ*ω* is the bandwidth of the cloak’s operating frequency^53^. If the operating frequency is greater than soliton’s spectral width, then the approximate cloak still works and is in principle experimentally feasible.

### Linear Background – Nonlinear Transform

While we have seen that transformation optics is robust to background nonlinearity, that case is easier to understand. The dynamics there are identical to nonlinear optics in Euclidean space, with the added linear transformation merely distorting the geodesics in fixed directions. When the transformation is nonlinear, then the geodesics can change with changing intensity. This makes, say, the combination of a nonlinear transform and the Kerr effect far harder to calculate. Instead, we shall now consider only a nonlinear transform and fix the background to be linear (upper right in Fig. 1). We can apply physical intuition to the nonlinear transform by taking inspiration from the study of effective gravitational fields via linear transformation optics^33,34^, where variations in the permittivity mimic the gravitational field produced by a mass distribution. In that case, the metric used must satisfy Einstein’s field equations

12 $${G}^{\mu \nu }={R}^{\mu \nu }-\frac{1}{2}R{g}^{\mu \nu }=\frac{8\pi G}{{c}^{4}}{T}^{\mu \nu }$$

where *R*_*μν*_ is the Ricci curvature tensor

13 $${R}_{\mu \nu }=2{{\rm{\Gamma }}}_{\mu [\nu ,\lambda ]}^{\lambda }+2{{\rm{\Gamma }}}_{\rho [\lambda }^{\lambda }{{\rm{\Gamma }}}_{\nu ]\mu }^{\rho }$$

14 $${{\rm{\Gamma }}}_{\nu \lambda }^{\mu }=\frac{1}{2}{g}^{\mu \rho }({g}_{\nu \rho ,\lambda }+{g}_{\lambda \rho ,\nu }-{g}_{\nu \lambda ,\rho }),$$

(where ${A}_{\mu \nu ,\rho }\equiv \tfrac{\partial {A}_{\mu \nu }}{\partial {x}^{\rho }}$, and *A*_[*μν*]_ ≡ (*A*_*μν*_ − *A*_*νμ*_)/2), *R* is the Ricci curvature scalar ${R}_{\mu }^{\mu }$, *G* is the gravitational constant, *c* is the speed of light, and *T*^*μν*^ is the stress-energy tensor. That is, a matter distribution is used to define *T*^*μν*^, which then defines *g*_*μν*_ via Eq. 12, thereby defining the equivalent $\epsilon$, *μ* via Eq. 1. However, relativity also predicts that energy and mass are equivalent (as in the famous ${\mathcal E} =m{c}^{2}$). As such, energy distributions can also define a stress-energy tensor and thereby produce a gravitational field^54^.

If the only source of energy is the electromagnetic field, then solutions to Eq. 12 are referred to as electrovacuum solutions. A material satisfying Eq. 3 with a metric transform obeying Eq. 12, then, will have a nonlinearity equivalent to the gravitational field produced by the electromagnetic field.

For a purely electromagnetic source, *T*^*μν*^ is

15 $$-{\mu }_{0}{T}_{\mu \nu }={F}_{\mu \alpha }{g}^{\alpha \beta }{F}_{\beta \nu }-{g}_{\mu \nu }\frac{1}{4}{F}_{\alpha \beta }{g}^{\beta \gamma }{F}_{\gamma \delta }{g}^{\delta \alpha }$$

where *F*_*μν*_ = ∂_*μ*_*A*_*ν*_ − ∂_*ν*_*A*_*μ*_ is the covariant electromagnetic field tensor and *A*^*α*^ is the electromagnetic four-potential $(\varphi ,\vec{A})$. For reference, contrast this with observations of a local, inertial observer, who will define

16 $${T}^{\mu \nu }=[\begin{array}{cc}U & {S}_{i}/c\\ {S}_{i}/c & {\sigma }_{ij}\end{array}]$$

where *U* is the energy density *U* = ($\epsilon$*E*^2^ + *μH*^2^)/2 = *E*_*i*_*D*^*i*^/2 + *B*_*i*_*H*^*i*^/2, $\vec{S}$ is the Poynting vector $\vec{E}\times \vec{H}$, and *σ* is the Maxwell stress tensor ${\sigma }_{ij}=\epsilon {E}_{i}{E}_{j}+\mu {B}_{i}{B}_{j}-\frac{1}{2}(\epsilon {E}^{2}+\mu {H}^{2}){\delta }_{ij}$. For both inertial and non-inertial observers with for a purely electromagnetic source, ${T}_{\mu }^{\mu }=0$ so *R* = 0 and our equations simplify. Additionally, we shall use the intuitive interpretation of a local observer to denote *T*^00^ with *U*(*E*), simplifying our notation.

**Figure 3:**
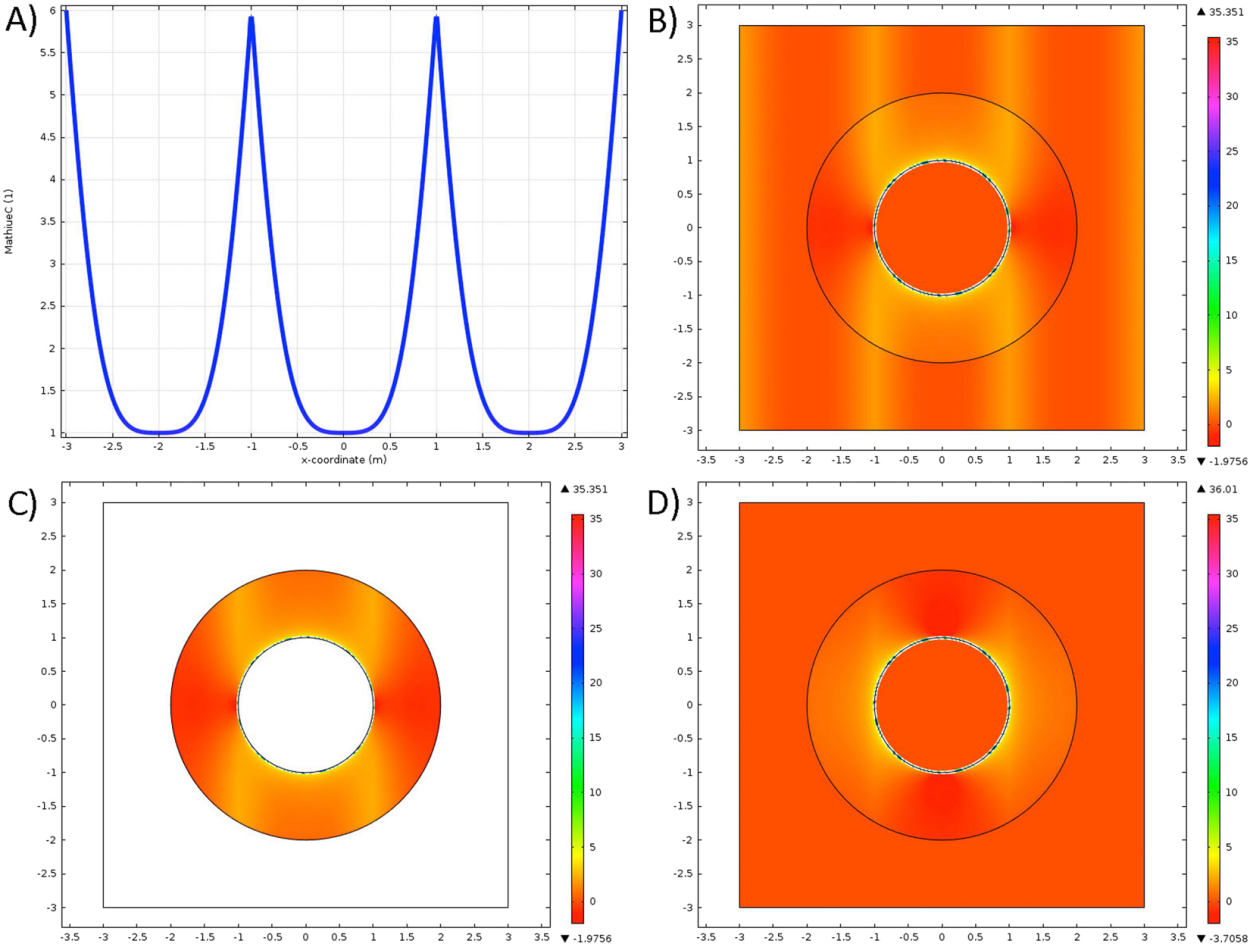
Electrovacuum cloak solution for a linear background. (**a**) Functional dependence of the metric vs position at constant field strength, using a periodic continuation to preserve a finite metric. (**b**) Corresponding value of ${\epsilon }_{xx}$, plotted on a log scale to handle singularity at *r* = *a*. (**c**) Log scale of ${\epsilon }_{xy}$, which is only non-zero within the cloak. (**d**) Log scale of ${\epsilon }_{yy}$.

To be more specific, we consider a plane wave solution $\vec{E}=|E|\,\cos (\omega t-\omega x/c)\hat{y}$ (note that other solutions exist for more realistic fields than a monochromatic plane wave, e.g.^55^, but are not as easily expressed in terms of the effective material parameters needed for transformation materials). In particular, our electromagnetic field is selected such that a local observer will detect a plane wave, which leads to ${A}_{\alpha }=\int \sqrt{f(u)}E(u)du{\delta }_{\alpha }^{2}$ where *f* is defined below in terms of the metric, *u* = *ω*(*t* − *x*/*c*). If our background is linear, then $U(E)={\epsilon }_{0}{E}^{2}$ (note that this is not the averaged energy, it retains space and time dependence), $\overrightarrow{S}=cU(E)\hat{x}$, and ${\sigma }_{ij}=-U(E){\delta }_{i}^{x}{\delta }_{j}^{x}$. We can then assume a metric of the form *g*_*μν*_ ≡ diag[−1, 1, *f*(*ct* − *x*), *f*(*ct* − *x*)] in Minkowski coordinates and use Eq. 12 to get

17 $$U(E)=\frac{f^{\prime\prime} }{f}-\frac{1}{2}{(\frac{f^{\prime} }{f})}^{2}=2h^{\prime\prime} /h,$$

defining *h* = *f*^2^. Using the identity $2\,{\cos }^{2}(\varphi )=1+\,\cos \,\mathrm{(2}\varphi )$, the stability condition *h* → 1 as |*E*| → 0, and the rotational symmetry (implying −|*E*| should give the same solution as |*E*|), gives

18 $$f={{\rm{M}}{\rm{a}}{\rm{t}}{\rm{h}}{\rm{i}}{\rm{e}}{\rm{u}}{\rm{C}}}^{2}(-\frac{|E{|}^{2}}{4{E}_{0}^{2}},\frac{|E{|}^{2}}{8{E}_{0}^{2}},\omega (ct-x)),$$

where MathieuC is the Mathieu cosine function (which, because the first term is negative, behaves closer to cosh than cosine) and ${E}_{0}=\omega c/\sqrt{4\pi G{\epsilon }^{\mathrm{(0)}}}$ is the natural electric field scale. Note that *G* only occurs in *E*_0_, and so an effective gravitational effect can be tuned by changing *E*_0_. In Fig. 3a, we plot *f*, where we’ve exploited the periodicity of Eq. 17 to create a periodic continuation of *f* (using the unmodified form results in an exponential growth of $\epsilon$). We now consider the composite transform *T*_*GR*,*C*_ = *T*_*C*_*T*_*GR*_, to create a cloaked region within this artificial relativistic medium. Using *f*, and Eqs 3 and 8 we calculate the components ${\epsilon }_{xx},{\epsilon }_{xy},{\epsilon }_{yy}$ and plot them in Fig. 3b–d. Notably, we do not plot *E* for this setup, as it is indistinguishable from the solution to a purely linear cloak. This is expected, given that the form of *E* was assumed in solving for *g*, but we can also show that Maxwell’s equations reduce to

19 $$\begin{array}{rcl}{\partial }_{tt}f{E}_{x} & = & {\partial }_{yy}{E}_{x}+{\partial }_{zz}{E}_{x}\\ {\partial }_{tt}{E}_{y} & = & {\partial }_{xx}{E}_{y}+{\partial }_{z}{f}^{-1}{\partial }_{z}{E}_{y}\\ {\partial }_{tt}{E}_{z} & = & {\partial }_{xx}{E}_{z}+{\partial }_{y}{f}^{-1}{\partial }_{y}{E}_{y},\end{array}$$

(*c* ≡ 1) which remain unchanged from linear Euclidean background from waves transverse to $\hat{x}$. A weaker test field, however, could detect the presence of the effective gravitational field if it propagated transversely to this electric field.

### Nonlinear Background – Nonlinear Transform

While the Mathieu cosine form of the nonlinear transform is helpful for illustrating the physical relevance of a nonlinear transform, an alternative formulation is preferable for developing materials prescriptions. In particular, it is preferable in nonlinear optics to know the dependence of the susceptibility as a Taylor series in *E*

20 $$P={\epsilon }_{0}\,\sum _{n=1}\,{\chi }^{(n)}{E}^{n}\equiv \sum _{n=0}\,({\epsilon }_{(n)}-{\epsilon }_{0}{\delta }_{n}^{0}){E}^{n+1}$$

to some finite order. In considering this problem, we shall once allow $\epsilon (E)$ to have an arbitrary nonlinearity, as that is most useful for design (bottom right of Fig. 1). Eq. 17 remains unchanged, save for a modification of *U*(*E*) to reflect the new value of $\epsilon (E)$, but the solutions can no longer be expressed in terms of analytic functions. Instead, we employ the Liouville-Neumann series technique to solve for *h*(*u*), where *u* = *ω*(*t* − *x*/*c*). That is, we consider a series expansion $h={\rm{\Sigma }}|E{|}^{2n}{h}_{n}(u)$, where ${h^{\prime} }_{n}\mathrm{(0)}=0$, ${h}_{n}\mathrm{(0)}={\delta }_{n}^{0}={h}_{0}$, and

21 $${h}_{n+1}(u)={\int }_{0}^{u}\,dw\,{\int }_{0}^{w}\,dvU(E(v)){h}_{n}(v)/2.$$

We truncate our solution *f* = *h*^2^ at 4^*th*^ order in *E*, as terms of that order and below are most relevant to nonlinear optics. However, truncation means that our solution takes the form *f*(*E* = |*E*| sin(*u*), *u*), as some terms have a more depend upon *u*^*m*^ (i.e. secular terms from nonlinear resonance) that cannot be factored without higher order terms (these likely correspond to the cosh dependence in the Mathieu solution).

This solution *f* gives the vacuum nonlinearity in a flat space-time. We now apply the cloaking transformation Eq. 8 in cylindrical coordinate to this metric and use Eq. 3 to get

22 $$\begin{array}{ccc}{\epsilon }_{ij}/\epsilon (E) & = & [\begin{array}{ccc}\frac{r-a}{r} & 0 & 0\\ 0 & \frac{r}{r-a} & 0\\ 0 & 0 & {(\frac{b}{b-a})}^{2}\frac{r-a}{r}\end{array}]\\  &  & +(f(E,u)-1)\,[\begin{array}{ccc}\frac{r-a}{r}\,{\cos }^{2}\,\theta  & -\,\cos \,\theta \,\sin \,\theta  & 0\\ -\,\cos \,\theta \,\sin \,\theta  & \frac{r}{r-a}\,{\sin }^{2}\,\theta  & 0\\ 0 & 0 & 0\end{array}]\end{array}$$

in transformed cylindrical coordinates (*μ*_*ij*_/*μ*(*E*) defined identically). To verify that this leaves Maxwell’s equations unchanged, it suffices to observe that this can also be written ${\epsilon }_{ij}(r,\theta ,E)={\epsilon }_{ij}^{(clk)}(r){\tilde{\epsilon }}_{ij}^{(nl)}(\theta ,E)$ (the nonlinear resonance should be modified to functions of *x*′ as they derive from *E*(*r*)), so ${\epsilon }^{(clk)}$ will reproduce Eq. 8, transforming Maxwell’s equations from a set of operators $L[r,{\epsilon }_{ij}^{(clk)}(r){\tilde{\epsilon }}_{ij}^{(nl)}(\theta ,E(r,\theta )),E(r,\theta )]$ to $L[r^{\prime} ,{\tilde{\epsilon }}_{ij}^{(nl)}(\theta ,E(r^{\prime} ,\theta )),E(r^{\prime} ,\theta )]$ as desired for a nonlinear cloaking transformation. Notice that the first term in Eq. 22 is the standard linear cloak (recall that *f*(*E* = 0) = 1 and the second is purely due to the vacuum nonlinearity. We can thus define ${\epsilon }_{ij}(E)/\epsilon (E)\equiv {\epsilon }_{ij}^{(l)}+(f(E,u)-\mathrm{1)}{\epsilon }_{ij}^{(nl)}$ for the linear and nonlinear coefficient matrices of Eq. 22. Multiplying by $\epsilon (E)$ and Taylor expanding in *E* thus gives

23 $$\begin{array}{rcl}{\epsilon }_{ij}(E) & = & {\epsilon }_{ij}^{(l)}{\epsilon }_{\mathrm{(0)}}+{\epsilon }_{ij}^{(l)}{\epsilon }_{\mathrm{(1)}}E+[{\epsilon }_{ij}^{(l)}{\epsilon }_{\mathrm{(2)}}{E}^{2}+{\epsilon }_{ij}^{(nl)}{\epsilon }_{\mathrm{(0)}}\tfrac{|E{|}^{2}{u}^{2}-{E}^{2}}{2{E}_{0}^{2}}]\\  &  & +[{\epsilon }_{ij}^{(nl)}{\epsilon }_{\mathrm{(1)}}\tfrac{\mathrm{24|}E{|}^{3}u+\mathrm{9|}E{|}^{2}E{u}^{2}-\mathrm{24|}E{|}^{2}E-13{E}^{3}}{18{E}_{0}^{2}}+{\epsilon }_{ij}^{(l)}{\epsilon }_{\mathrm{(3)}}{E}^{3}]\\  &  & +[{\epsilon }_{ij}^{(l)}{\epsilon }_{\mathrm{(4)}}{E}^{4}+{\epsilon }_{ij}^{(nl)}({\epsilon }_{\mathrm{(2)}}\tfrac{\mathrm{3|}E{|}^{4}{u}^{2}-\mathrm{5|}E{|}^{2}{E}^{2}+\mathrm{4|}E{|}^{2}{E}^{2}{u}^{2}-2{E}^{4}}{8{E}_{0}^{2}}+\tfrac{{\epsilon }_{\mathrm{(1)}}^{2}}{{\epsilon }_{\mathrm{(0)}}}\tfrac{12u|E{|}^{3}E-\mathrm{12|}E{|}^{2}{E}^{2}-2{E}^{4}}{9{E}_{0}^{2}})\\  &  & +{\epsilon }_{ij}^{(nl)}({\epsilon }_{\mathrm{(0)}}\tfrac{\mathrm{5|}E{|}^{4}{u}^{4}+\mathrm{3|}E{|}^{4}{u}^{2}+3(11-8u-6{u}^{2})|E{|}^{2}{E}^{2}-24u|E{|}^{3}E\sqrt{|E{|}^{2}-{E}^{2}}-3{E}^{4}}{48{E}_{0}^{4}})]\\  &  & +O({E}^{5}\mathrm{).}\end{array}$$

Notice that, although *E*_0_ was originally defined in terms of the constant *G*, it is the only place that such constant enters into the transformed material equation. Thus, we are free to redefine *E*_0_ as any effective scale for the electric field strength, rather than the scale prescribed by Eq. 12. That is, we can use transformation optics to model a nonlinear gravitational field with arbitrary strength *E*_0_(*G*_*eff*_).

#### Realization

Equation 23 includes nonlinear effects up to fourth order, which is considerably higher than most applications for nonlinear optics. Although effects like four wave mixing explicitly rely upon these fourth order nonlinearities (hence their inclusion in equation 23), it is far more common to retain only the lowest order non-trivial nonlinearity. Assuming our background lacks an ${\epsilon }_{\mathrm{(1)}}$ term, which is trivially decoupled from the gravitational nonlinearity, the first non-trivial term is the secondorder term:

24 $${\chi }_{ijkl}^{\mathrm{(3)}}={\epsilon }_{ij}^{(l)}{\varepsilon }_{\mathrm{(2)}}{E}^{2}+{\epsilon }_{ij}^{(nl)}{\epsilon }_{\mathrm{(0)}}\frac{|E{|}^{2}{u}^{2}-{E}^{2}}{2{E}_{0}^{2}}\mathrm{.}$$

However, the *kl* dependence of *χ*^(3)^ is left implicit here and the *u*^2^ dependence gives a non-stationary nonlinearity. This issues can be corrected to lowest order by making the approximation *u* ≈ sin *u* to get |*E*|^2^*u*^2^ − *E*^2^ ≈ |*E*|^2^ − 2*E*^2^. If we interpret the direction of our input field as fixed, this gives

25 $${\chi }_{ijkl}^{(3)}={\epsilon }_{ij}^{(l)}{\epsilon }_{(2)}{E}^{2}{\delta }_{kl}{\delta }_{ly}+{\epsilon }_{ij}^{(nl)}{\epsilon }_{(0)}\frac{1}{2{E}_{0}^{2}}(|E{|}^{2}-2{E}^{2}{\delta }_{ly}){\delta }_{kl}.$$

On the other hand, recalling that our original choice of the direction of $\vec{E}$ was arbitrary within an isotropic background gives us

26 $${\chi }_{ijkl}^{\mathrm{(3)}}={\epsilon }_{ij}^{(l)}{\epsilon }_{\mathrm{(2)}}{E}^{2}{\delta }_{kl}+{\epsilon }_{ij}^{(nl)}{\epsilon }_{\mathrm{(0)}}\frac{1}{2{E}_{0}^{2}}(|E{|}^{2}-2{E}^{2}){\delta }_{kl}\mathrm{.}$$

Either form of *χ* gives the simplest model of the gravitational nonlinearity, which can be seen as a more complex form of Kerr nonlinearity.

#### Applications

Given the complexity present in even the simplest possible realizations of this gravitational nonlinearity, it is unlikely that these nonlinear gravitational transformations will find experimental applications in the near future. However, these results hold an important theoretical application – namely the elucidation of the correspondence between nonlinear optical media and electrovacuum gravitational fields. This implies that difficult problems in general relativity can be mapped to equivalent problems in nonlinear optics, where approximations as to the effect of nonlinearity are more robust. Simultaneously, results in nonlinear optics can now be interpreted as effectively gravitational, providing an interesting intuitive picture of photon-photon interactions not normally considered in the standard treatment.

### Transformation Media Extension

Before considering our final example, it is worth stepping back and considering how these nonlinear transformation optics techniques could be extended to other forms of transformation media. Acoustics is by far the easiest generalization, as there are straightforward mappings from transformation optics to transformation acoustics^17^. Heat transport and diffusion are more difficult, however. While the introduction of field dependence to the already established thermal transformation^19^ holds – i.e. that

27 $${\kappa }^{ij}/{\kappa }_{0}(T)={g}^{ij}(T)\sqrt{-g(T)}$$

28 $$\rho {c}_{p}/{\rho }_{0}(T){c}_{p0}(T)=\sqrt{-g(T)}$$

−is valid, the diffusion equation is not Lorentz invariant and therefore is not a valid equation for the relativistic interpretation. Thus, while transformation materials is applicable to nonlinear heat transport, it cannot be interpreted in terms of effective gravitational fields. However, because transformation diffusion is defined for an isotropic background *κ*_0_, with all anisotropy arising from the transformation, a further interpretation is plausible. Both the background nonlinearity and isotropic nonlinear transform control the speed of diffusion at a given temperature, whereas the anisotropic aspect controls the preferential direction of diffusion as a function of temperature.

So, for our final example we consider heat transport within a Debye solid (*κ* ∝ (*T*/*T*_0_)^3^, *c*_*p*_ ∝ (*T*/*T*_0_)^3^, *ρ* = *ρ*_0_/(1 + *αT*) ≈ *ρ*_0_, where *T*_0_ is the Debye temperature, and *α* is thermal expansivity (*O*(10^−5^/*K* for a solid)). As the nonlinear transform in this case is an arbitrary *g*(*T*) that does not satisfy Eq. 12, we consider a “phase transition” transform

29 $$\lambda (r,T)=\frac{{\lambda }_{L}(r)+{\lambda }_{H}(r)}{2}+\frac{{\lambda }_{H}(r)-{\lambda }_{L}(r)}{2}\,\tanh \,\frac{T-{T}_{tr}}{{T}_{{\rm{\Delta }}}},$$

where *λ* = *ρc*_*p*_,*κ*_*rr*_ or *κ*_*θθ*_, *λ*_*L*(*H*)_ are the low (high) temperature transformed parameters, *T*_*tr*_ is the transition temperature, and *T*_Δ_ is the range of the intermediate zone. In particular, we want a cloak for high temperatures (Eq. 8, *T* > *T*_*tr*_) and a concentrator for low temperatures. For a concentrator’s transformation a central *r* < *R*_2_ is shrunken down to *r* < *R*_1_ where *R*_1_ < *R*_2_. A second region, *R*_2_ < *r* < *R*_3_ is then stretched out to accommodate the shrunken central region. Contrast with equation 8, where a single point is expanded to a finite diameter, leaving a hole in the center and only requiring one transformed region *a* < *r* < *b*. Typically^20^, the concentrator uses a radially linear mapping

30 $$r^{\prime} =\{\begin{array}{ll}\frac{{R}_{1}}{{R}_{2}}r & 0 < r^{\prime}  < {R}_{1}\\ \frac{{R}_{3}-{R}_{1}}{{R}_{3}-{R}_{2}}r+\frac{{R}_{1}-{R}_{2}}{{R}_{3}-{R}_{2}}{R}_{3} & {R}_{1} < r^{\prime}  < {R}_{3}\end{array}$$

which can be unified with the cloaking transform of equation 8 by identifying *R*_1_ = *a*, *R*_3_ = *b* and leaving *R*_1_ < *R*_2_ < *R*_3_ a free parameter (i.e. the second region in equation 30 is defined over the same domain as equation 8. Making this identification, we can define our high temperature (cloak) phase to have the parameters *λ*_*H*_

31 $$\begin{array}{ccc}\frac{\rho (T){c}_{p}(T)}{{\rho }_{0}(T){c}_{p0}(T)} & = & {(\frac{{R}_{3}}{{R}_{3}-{R}_{1}})}^{2}\frac{r-{R}_{1}}{r}\\ \frac{{\kappa }_{rr}(T)}{{\kappa }_{0}(T)} & = & \frac{r-{R}_{1}}{r}\\ \frac{{\kappa }_{\theta \theta }(T)}{{\kappa }_{0}(T)} & = & \frac{r}{r-{R}_{1}}.\end{array}$$

For the same annular region the low temperature (concentrator) phase has parameters *λ*_*L*_

32 $$\begin{array}{ccc}\frac{\rho (T){c}_{p}(T)}{{\rho }_{0}(T){c}_{p0}(T)} & = & {(\frac{{R}_{3}-{R}_{2}}{{R}_{3}-{R}_{1}})}^{2}(1+\frac{{R}_{3}}{r}\frac{{R}_{2}-{R}_{1}}{{R}_{3}-{R}_{2}})\\ \frac{{\kappa }_{rr}(T)}{{\kappa }_{0}(T)} & = & 1+\frac{{R}_{3}}{r}\frac{{R}_{2}-{R}_{1}}{{R}_{3}-{R}_{2}}\\ \frac{{\kappa }_{\theta \theta }(T)}{{\kappa }_{0}(T)} & = & {(1+\frac{{R}_{3}}{r}\frac{{R}_{2}-{R}_{1}}{{R}_{3}-{R}_{2}})}^{-1},\end{array}$$

while the central circle (*r* < *R*_1_) has parameters

33 $$\begin{array}{ccc}\rho (T){c}_{p}(T)/{\rho }_{0}(T){c}_{p0}(T) & = & {(\frac{{R}_{2}}{{R}_{1}})}^{2}\\ {\kappa }_{rr}(T)/{\kappa }_{0}(T) & = & {\kappa }_{\theta \theta }(T)/{\kappa }_{0}(T)=1\end{array}$$

for all temperatures (technically, the properties of the high temperature, cloaked phase are free parameters since they are not defined by equation 8, but in practice it is simpler to use the same thermal dependence as the background and make everything consistent). Notice that for both phases ${\kappa }_{rr}(T){\kappa }_{\theta \theta }(T)={\kappa }_{0}^{2}(T)$, which is helpful since many discretized thermal metamaterial implementations of the thermal cloak/concentrator rely upon this symmetry being preserved^19,20^. It is thus conceivable to consider building such thermal metamaterials necessary for the cloak-concentrator out of a binary composite where one material undergoes a phase transition at a specific temperature. For concreteness, we define the overall form of the cloak-concentrator’s material parameters (including both spatial and thermal dependance in the annular region)

34 $$\begin{array}{ccc}{\textstyle \tfrac{\rho (T){c}_{p}(T)}{{\rho }_{0}(T){c}_{p0}(T)}} & = & {\textstyle \tfrac{{({\textstyle \tfrac{{R}_{3}-{R}_{2}}{{R}_{3}-{R}_{1}}})}^{2}(1+{\textstyle \tfrac{{R}_{3}}{r}}{\textstyle \tfrac{{R}_{2}-{R}_{1}}{{R}_{3}-{R}_{2}}})}{2}}(1-\,\tanh ({\textstyle \tfrac{T-{T}_{tr}}{{T}_{{\rm{\Delta }}}}}))\\  &  & +{\textstyle \tfrac{{({\textstyle \tfrac{{R}_{3}}{{R}_{3}-{R}_{1}}})}^{2}{\textstyle \tfrac{r-{R}_{1}}{r}}}{2}}(1+\,\tanh ({\textstyle \tfrac{T-{T}_{tr}}{{T}_{{\rm{\Delta }}}}}))\\ {\textstyle \tfrac{{\kappa }_{rr}(T)}{{\kappa }_{0}(T)}} & = & {\textstyle \tfrac{1+{\textstyle \tfrac{{R}_{3}}{r}}{\textstyle \tfrac{{R}_{2}-{R}_{1}}{{R}_{3}-{R}_{2}}}}{2}}(1-\,\tanh ({\textstyle \tfrac{T-{T}_{tr}}{{T}_{{\rm{\Delta }}}}}))\\  &  & +{\textstyle \tfrac{1}{2}}{\textstyle \tfrac{r-{R}_{1}}{r}}(1+\,\tanh ({\textstyle \tfrac{T-{T}_{tr}}{{T}_{{\rm{\Delta }}}}}))\\ {\textstyle \tfrac{{\kappa }_{\theta \theta }(T)}{{\kappa }_{0}(T)}} & = & {\textstyle \tfrac{1}{2}}{(1+{\textstyle \tfrac{{R}_{3}}{r}}{\textstyle \tfrac{{R}_{2}-{R}_{1}}{{R}_{3}-{R}_{2}}})}^{-1}(1-\,\tanh ({\textstyle \tfrac{T-{T}_{tr}}{{T}_{{\rm{\Delta }}}}}))\\  &  & +{\textstyle \tfrac{1}{2}}{\textstyle \tfrac{r}{r-{R}_{1}}}(1+\,\tanh ({\textstyle \tfrac{T-{T}_{tr}}{{T}_{{\rm{\Delta }}}}})).\end{array}$$

**Figure 4:**
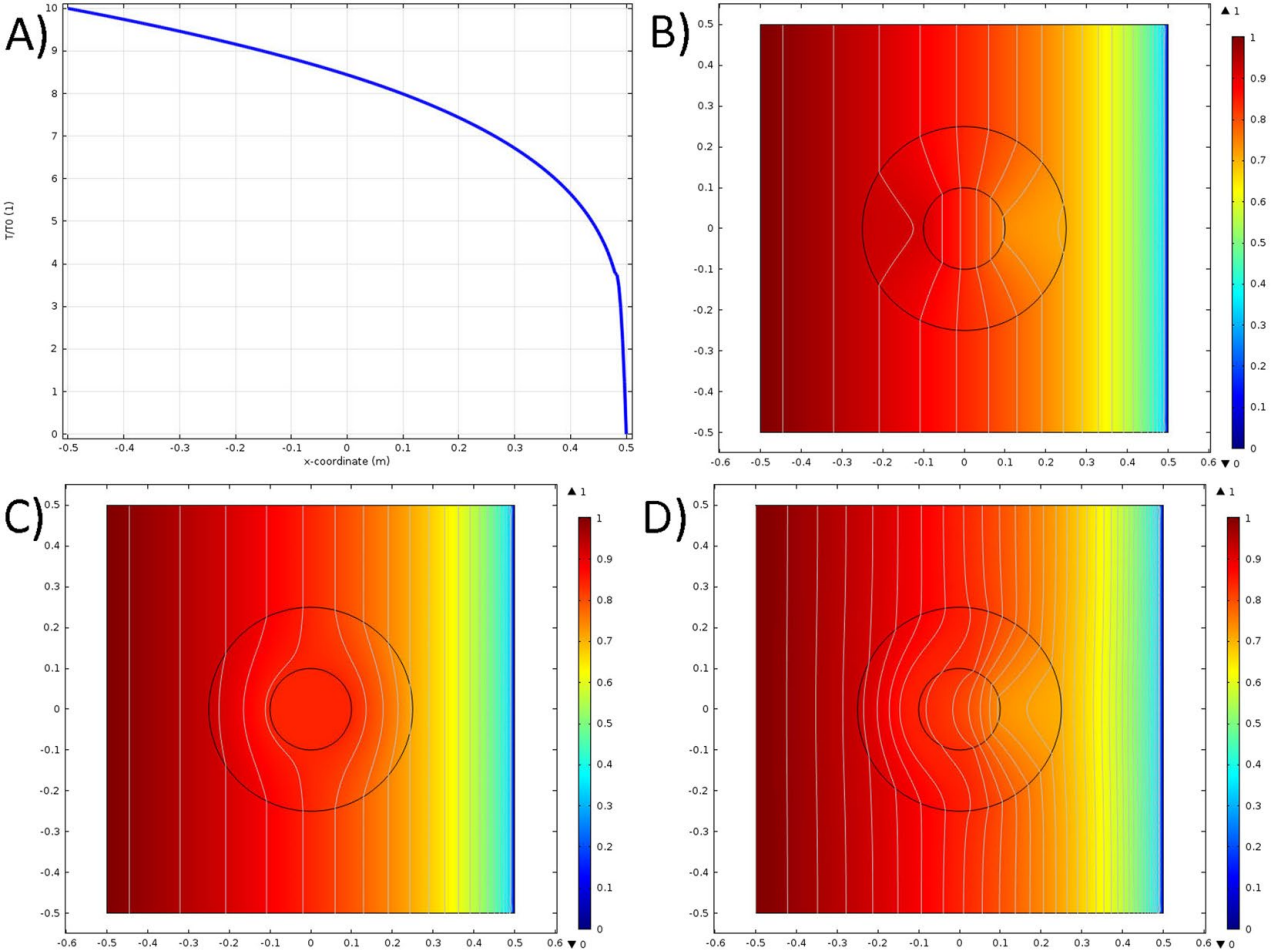
Transformation diffusion of a Debye solid with switchable nonlinear concentrator/cloak transform. Steady state plots with *T*(*x* = −*L*/2) = *T*_1_, *T*(*x* = *L*/2) = 0. Note that isotherms (grey lines) are not evenly spaced due to Debye nonlinearity. (**a**) Far field temperature distribution. (**b**) Low temperature, *T*_1_ = 0.1*T*_0_, concentrator. (**c**) High temperature, *T*_1_ = 10*T*_0_, cloak. (**d**) Transitional, half cloak, half concentrator.

Note that for $T\gg {T}_{tr}$ equation 34 converges to equation 31, for $T\ll {T}_{tr}$ equation 34 converges to equation 32, and *T* ≈ *T*_*tr*_ is equal to the average of equations 31 and 32 but switches quickly to either the high or low temperature limit when $|T-{T}_{tr}|\mathop{ > }\limits_{ \tilde {}}{T}_{{\rm{\Delta }}}$. This behavior is confirmed by COMSOL simulations, where the far field temperature distribution in the nonlinear background is unchanged by the presence of the transformation materials (Fig. 4a) and that the low (Fig. 4b) or high (Fig. 4c) temperature cases work as a thermal concentrator or cloak of the Debye solid. More interesting is the intermediate case when *T*(*x* = 0) ≡ *T*_*tr*_ ≈ 8.4*T*_0_, i.e. when transition temperature isotherm bisects the annulus. In this regime the device acts like a cloak for $x\mathop{ < }\limits_{ \tilde {}}0$ and a concentrator for $x\mathop{ > }\limits_{ \tilde {}}0$, Fig. 4d. A careful examination of *x* ≈ 0 reveals that neither the device is neither a cloak or a concentrator close to the transition temperature, with isotherms shifting to reflect this compromise regime. In particular, the thermal gradient within the central domain neither vanishes (as in the cloak) or a constant (as in the concentrator, note the isotherms in Fig. 4b), but curved towards the low temperature phase. Moreover, within cloak-concentrator there is a clear asymmetry to the slope of the isotherms for *x* > 0 vs *x* < 0 in the high and low temperature phases but not in the compromise region (the slope of some isotherms even oscillates near *x* = 0). However, this intermediate region could be made extremely narrow in practice, as it corresonds to a coexistance of the high and low temperature phases of the thermal metamaterial and conventional materials generally have unambiguous state for first order phase transition.

#### Applications

With this functionality we have created a device which excludes heat flux at high temperatures and attracts it at low temperatures, thereby acting as an effective thermal regulator. In addition, there is ability of the cloak (equation 31) and concetrator (equation 32) designs to function correctly when embedded within temperature dependent materials is a non-trivial improvement of the functionality of thermal transformation materials, as all realistic materials will possess some temperature dependence to their parameters. It is also worth noting that even physical parameters unrelated to thermal transport will generally possess some form of temperature dependence, and therefore that the incorporation of designs for transformation materials with non-trivial temperature dependent backgrounds or transformations could greatly improve the versatility of transformation materials. Similar arguments can be made for materials with dependence upon pressure, electric, magnetic, and electromagnetic fields, albeit for narrower ranges of materials.

#### Realization

Since most materials possess some degree of temperature dependence to their thermal properties, the realization of the cloak-concentrator seems fairly straightforward and is perhaps the most easily achieved of all the examples discussed in this work. To work through how it might be realized, however, we shall make several approximations. In particular, as our goal is the simplest experimental realization, we shall consider the design of the bilayer cloak^21^ as our starting point. This design, which used a pair of annular regions of homogeneous, isotropic material to create a thermal cloak, explicitly focused upon the steady state. This implied that the heat capacity could be completely neglected, as engineering the conducitivity was all that was required. More subtly, the bilayer cloak also assumed that the superposition holds in its derivation, and therefore only works rigorously for materials without temperature dependence. As such, we shall neglect the temperature dependence of the background and assume that the materials in our cloak-concentrator are approximately independent of temperature except for near their respective (and possibly distinct) transition temperatures, where the operation of the device breaks down. Making these assumptions turns the problem into a two-fold one: first a bilayer concentrator must be designed to match the implementation of the bilayer cloak. And second there must be a pair of materials which can, after undergoing phase transitions, meet the thermal conductivity requirements in each layer. To address the first part, consider the logic of the bilayer cloak – to minimize the thermal flux in the center the inner ring’s conducitivity was set as close to zero as possible and the outer ring’s conducitivity was then adjusted to prevent any scattering at the outer edge of the cloak. For the concentrator a similar requirement holds, the outer layer must prevent scattering, but the inner layer’s conductivity must be adjusted to maximize the heat flux in the interior (which is filled with the same material as the background). Numerically optimizing the analytic solution to Laplace’s equation with these constraints gives properties as laid out in Table 1, which enhances the heat flux in the concentrator regime by 8 percent. Note that the temperature dependence of the conductivities in each layer are opposed to each other, with the inner layer’s conducitivity falling with temperature and the outer layer’s conductivity rising with rising temperature. This is a non-trivial temperature dependence, but not outside of the realm of experimental possiblity – notably thermal diodes have been implemented using pairs of materials with similar thermal properties. As such, the thermal cloak-concentrator is likely the ideal candidate for the experimental verification of nonlinear thermal transformations, particularly if the constraint of *κ*_*i*_ → 0 for the cloak is relaxed (i.e. an approximate cloak design is used.

**Table 1:** Properties of the temperature independent cloak-concentrator.

Layer	Radius [cm]	*κ*_*Cloak*_ [W/mK]	*κ*_*Concentrator*_ [W/mK]
Interior	6	1	1
Inner	9	0.01304	1.558
Outer	10.2	4.261	0.6486
Exterior	N/A	1	1

Cloak parameters taken from ref.^21^, with concentrator conductivity chosen to optimize heat flux given the geometry.

## Conclusions

In summary, we have developed a formalism for understanding transformation materials in nonlinear media and undergoing nonlinear transformations, and shown how this formalism can be applied to soliton transport (with applications in hiding objects from nonlinear detection protocols or shielding rogue waves), effective gravitational fields (with applications in modeling relativistic effects in nonlinear optical materials), and thermal management (with applications in controlling heat flow in realistic, thermally nonlinear materials or incorporating temperature dependent parameters in arbitrary transformation media devices). It is therefore possible to use nonlinear transformation media to model a wider variety of transport phenomena than have been previously considered. Furthermore, the constitutive relations that we have derived in equations 3, 23, 27 and 34 can be used for a wider variety of transformations than just the cloaking transformation (e.g. concentrators, rotators, camoflague, etc.).

### Additional Applications

Given the incorporation of frequency dependent material parameters in the metamaterial realizations of transformation optics^11^ and the development of nonlinear metamaterials^56^, the incorporation of nonlinear optical transformations presents the opportunity for a wider variety of functional materials in this framework. Wave mixing, for example, could be used to generate additional field components at a frequency several times the incident wave’s. If a metamaterial has different resonant effects at these frequencies, it could (say), screen the incident wave while concentrating the nonlinear contribution. This could be accomplished with metamaterials whose parameters are quite similar to equation 34, except that the dependence upon (*T* − *T*_*tr*_)/*T*_Δ_ would go to a dependence upon (*ω* − *ω*_*tr*_)/*ω*_Δ_. Or it could change the relative field concentration within different regions (e.g. via a concentrator or its inverse, a dilutor), thereby controlling the effective strength of the local nonlinearity (increasing the effective nonlinearity in high fields regions and decreasing it in low field regions), which could alternatively aide in the observation of novel nonlinear effects or prevent the occurence of unwanted nonlinearities. This approach is quite similar to that applied by certain classes of nonlinear metamaterials, which rely upon concentrating the electromagnetic field on regions of strong nonlinearity^57^ or improving the efficiency of nonlinear conversion (e.g. via phase matching)^58^. Soliton formation^59^ could also be promoted, say by counteracting an excessive dispersion or nonlinearity in the external medium (an alternative approach to that adopted by^60^).

### General Realizations

In principle, any of these applications could be implemented with the utilization of nonlinear metamaterials, which have so far developed independently of advances in transformation materials, being more focused upon the creation of arbitrary strength nonlinearities. Nonlinear metamaterials can be produced by adding nonlinear elements to a linear metamaterials design^61^ or embedding such designs in nonlinear backgrounds^62^, or introducing intrinsic structural nonlinearities to linear materials (e.g. magnetoelastic structural reconfiguration)^63^. Such nonlinear metamaterials have already been used for elementary soliton steering applications^60^. Note that all of these techniques are reviewed in more detail in Lapine *et al*.^56^ and the references therein. The combination of frequency mixing and resonance is not necessarily a fatal flaw to these designs either. While most of the devices considered here (cloaks, concentrators, etc.) rely upon a tailored metamaterial resonance for their implementation, the potential existence of waves at multiple, distant frequencies can be accounted for. In particular, as the metamaterial resonance is tied to the length scale of the structuring, the incorporation of multiple length scale would allow for additional resonances. Thus, a cloak designed for a frequency doubling application should be designed with two resonant lengt scales (the doubled frequency corresponding to half the length). While that complicates the fabrication, it should be achievable so long as the specification application is well-defined in advance. Generally, nonlinear transformations can increase the versatility of frequency-dependent phenomena in metamaterials and transformation materials.
